# Chiropractic website claims related to non-musculoskeletal conditions: a cross-sectional study

**DOI:** 10.1186/s12998-021-00397-y

**Published:** 2021-09-22

**Authors:** Guillaume Goncalves, Philippe Fleuriau, Charlène Cheron, Mathieu Picchiottino, Sylvain Pigeon, Rikke Krüger Jensen

**Affiliations:** 1 Société Franco-Européenne de Chiropraxie, 59700 Marcq-en-Baroeul, France; 2 Private Practice, 14000 Caen, France; 3 Department of Sport Science and Clinical Biomechanics, University of Southern Denmark, Campusvej 55, 5230 Odense M, Denmark; 4 Chiropractic Knowledge Hub, Campusvej 55, 5230 Odense M, Denmark; 5 Faculty of Allied Health & Wellbeing, University of Central Lancashire (UCLan), Preston, UK

**Keywords:** Chiropractor, Website, Non-musculoskeletal, Advertising

## Abstract

**Background:**

Chiropractors frequently use spinal manipulation therapy as a treatment modality in the management of musculoskeletal (MSK) conditions such as spinal pain and sometimes in the treatment of non-MSK disorders. The latter is not supported by evidence. This study aimed to investigate the extent of non-MSK website claims from French chiropractors to assess if websites were aligned with current recommendations on evidence-based practice.

**Methods:**

This was a cross-sectional study of a representative sample of French chiropractors. Information on non-MSK conditions was collected from chiropractic professional websites by two independent observers in June 2020. For each non-MSK condition, it was noted whether a clarifying explanation justifying the mentioning of the condition was available. In addition, data on professional association affiliation status, country of education, years since graduation, and special clinical focus (e.g., seniors, children) were collected.

**Results:**

A total of 287 randomly selected websites were included in the study corresponding to 22% of all chiropractors registered in France. One or more of 42 different non-MSK conditions were identified on 231 websites, of which 5 (2.2%) provided a clarifying explanation. 226 (79%) websites mentioned a non-MSK condition without a clarifying explanation. The non-MSK conditions most often mentioned were sleep problems, constipation/digestive problems, unease/discontent in children and menstrual cramps/pains. A larger proportion of the websites advertising treatment for non-MSK disorders was found among those claiming a special clinical focus on infants/children, seniors, pregnant women or athletes compared to those that did not. Also, a larger proportion of chiropractors who were affiliated with a professional association were advertising treatment for non-MSK disorders compared to those who were not affiliated.

**Conclusions:**

Eight out of ten chiropractic websites mentioned one or more non-MSK diagnoses or symptoms. It is unclear if this reflects inaccurate communication or if treatments for non-MSK conditions are provided by the chiropractors.

**Supplementary Information:**

The online version contains supplementary material available at 10.1186/s12998-021-00397-y.

## Background

Health care providers are expected to adhere to an evidence-based practice approach and to respect ethical and legal requirements when providing information to patients and the general public. Practicing according to an evidence-based model includes integrating clinical expertise and patient preferences with the best available evidence from systematic research [1]. The latter has been defined as: “… clinically relevant research […] especially from patient centred clinical research into the accuracy and precision of diagnostic tests (including the clinical examination), the power of prognostic markers, and the efficacy and safety of therapeutic, rehabilitative, and preventive regimens” [1].

The term ‘manual therapies’ represents a group of therapeutic modalities that have been investigated for their effectiveness on both musculoskeletal (MSK) and non-MSK disorders. For example, clinical guidelines and recent meta-analyses recommend spinal manipulation therapy (SMT) as a safe alternative to other conservative treatments for spinal pain [2–6]. However, an extensive systematic review from 2021 found no evidence of an effect of SMT on the management of non-MSK disorders including childhood asthma, hypertension, and primary dysmenorrhea [7]. This review confirmed previous conclusions from systematic reviews on the topic [8–10].

Chiropractors frequently use SMT as a treatment modality in the management of MSK conditions such as spinal pain, and in some cases in the treatment of non-MSK disorders [11]. In some countries (e.g., Denmark, Switzerland) chiropractors are recognized as a health care profession and fully integrated into the national health care system. In other countries, the profession is only partly integrated into the health care system, which is the case with the French chiropractic profession.

Chiropractic was recognized in France in 2002 [12, 13] and is taught in a private college with an evidence-based approach. The education is accredited by the French Ministry of Health and the Ministry of Higher Education but in contrast to for example Denmark and Switzerland, it does not have an undergraduate university connection [14]. According to French legislation, all French chiropractors are authorized to prevent and treat neuro-MSK disorders and their consequences [12, 13], and are obligated to be guided by an evidence-based approach in their practice [14]. In contrast to physiotherapists and medical doctors, chiropractic treatment is not reimbursed by health care state insurance in France.

The French Chiropractic Association (AFC) works for improved integration of French chiropractors into the national health care system by supporting an evidence-based practice (i.e., not advertising unsubstantiated claims). However, this policy is not supported by all French chiropractors. Therefore, an association may exist between not being a member of AFC and advertising non-musculoskeletal claims.

French legislation regarding the possibility for a medical doctor to communicate on the internet [15] advises the doctor to do so with caution and based on evidence-based information. It is against the law to (i) mislead patients toward non-useful health care and (ii) be prejudicial for the reputation of the medical profession. It is also against the law for a medical doctor to present preliminary data as validated information on the clinic website.

Chiropractors in France do not have this kind of requirement yet. However, as they aim to improve their integration into the French health care system, chiropractors would be expected to follow similar legislation. By comparison, Danish chiropractors have a university-based education and are integrated into the national health care programme. Yet, surprisingly, a cross-sectional study investigating website claims by Danish chiropractors found that 26% mention non- MSK conditions [16]. Also, non-musculoskeletal claims on chiropractic websites have been identified in studies from Canada [17, 18], New Zealand [19], and South Africa [20]. These studies have reported that attention deficit disorders were mentioned on 25% of New Zealand and 29% on Canadian websites; allergy/asthma on 33% of Canadian and 35–39% of New Zealand websites; and constipation/digestive problems on 16% of South African websites.

The French chiropractic education is not university-based and chiropractic services are not fully integrated into the national health care programme (e.g., no reimbursement from national insurance, no established cooperation with medical doctors and physiotherapist/*kinésithérapeutes*)—, and it is unknown to what extent French chiropractors claim to prevent or treat non-MSK disorders. Therefore, this study aimed to investigate the content of websites claims from French chiropractors to access if they were aligned with current recommendations on evidence-based practice.

Our specific objectives were to investigate:

1. If French chiropractors offer treatment for non-MSK conditions on their websites, and if so, how common this is and for which types of conditions they offer treatments.
2. Whether the professional association affiliation, the country of education, or the years since graduation were associated with the content of their websites.

## Methods

### Design

An observational cross-sectional study.

### Setting

Data were collected from websites owned by French chiropractors. It is mandatory for a chiropractor practicing in France to be registered with a state-administered regional health agency. A list of all registered chiropractors who practice in France was obtained through the registration body in May 2020.

For practical reasons, it was not possible to assess all chiropractic websites in France, therefore a representative sample was extracted. For the results to be representative of the whole of metropolitan France (i.e., inside Europe’s geographical borders), the sampling was stratified according to the population of each region. A sample of chiropractors from each of the 13 regions was randomly selected using a random number generator [21]. The chiropractors’ websites were then searched on the two first pages on a *Google* search using the equation: “[First name] [Last name] [chiropracteur]”.

If a chiropractic clinic did not have a website or if the website was already included in the sample (duplicate)—due to more than one chiropractor working in the same clinic—another chiropractic clinic (from the same region) was randomly selected using a random number generator [21]. Professional websites containing only basic information (i.e., chiropractors’ contact information, testimonials, and eventually a link to make an appointment) were not considered eligible. All sampling sessions were performed between June 29th, 2020, and July 4th, 2020.

### Data collection

Prior to data collection, a data collection tool used in a similar Danish study [16] was translated into French by GG and a senior researcher bilingual in Danish and French (CLY). The English version of the data collection tool is available as Additional file 1. Two training sessions with all five members of the collection group (GG, PF, CC, MP, SP) were carried out. During the first training session, each item of the data collection tool was discussed among the members and one website was evaluated as an example. At the end of this session, all members of the collection group were asked to individually evaluate a small sample of websites (n = 5). During the second training session, the results of the evaluated websites were discussed, and consensus was reached on all items.

Websites were divided into four groups, each assessed by one of the four pairs of data collectors (GG/PF, GG/CC, GG/MP, GG/SP). Each person independently examined the sampled websites and extracted data between July 27th, 2020, and December 31st, 2020. In case of disagreement, a consensus was reached by discussion within the pair or—if necessary—after consulting another member of the data collection group.

To ensure that all relevant information was retrieved, it was possible to add new variables if they emerged during data collection. Website information was recorded as present if mentioned in the main text on the website or in a drop-down menu. If the chiropractic website provided a link to another website describing a disorder, it was recorded as not present unless the chiropractic website clearly mentioned that the link provides information on the conditions treated in the clinic.

### Variable of interest

#### Chiropractors

To enable an equally distributed geographical representation, the sampling was stratified according to region i.e., Region (1) *Auvergne Rhône Alpes*; Region (2) *Haut de France*; Region (3) *Provence Alpes Côtes d’Azur*; Region (4) *Grand Est*; Region (5) *Occitanie*; Region (6) *Normandie*; Region (7) *Nouvelle Aquitaine*; Region (8) *Centre Val de Loire*; Region (9) *Corse*; Region (10) *Bourgogne Franche Comté*; Region (11) *Pays de la Loire*; Region (12) *Bretagne*; Region (13) *Ile de France*.

Information on the chiropractor’s affiliation to the French Chiropractic Association (through the professional association registry) was collected as well as the country of education and year of graduation (through website content and/or professional registry). The information was collected on all randomly selected chiropractors even if they were not included due to a missing website. This enabled comparison between included and non-included chiropractors from all the selected chiropractors.

#### Patient groups

It was registered if the chiropractors had a special interest in treating specific patient groups. This information was recorded and categorised as ‘infant’, ‘children’, ‘seniors’, ‘pregnant women’, ‘athletes’ and ‘disabled people’. After the two training sessions, the data collection group (GG, PF, CC, MP, SP) decided to add ‘musicians’ as a new specific patient group to the previously used data collection tool. ‘Animals’ was also added as a special interest.

#### Diagnoses and symptoms of non-musculoskeletal conditions

Information on non-MSK conditions from the websites was noted in the French data collection tool. If a diagnosis or symptom did not fit any of the predefined categories a new category was added. Predefined categories included both specific diagnoses such as ‘High blood pressure’ or ‘Allergy’ and more general symptoms as for example ‘Abdominal pain’.

For each non-MSK condition, it was recorded whether a ‘clarifying explanation’ was available. These explanations were assessed and categorised for compliance with the chiropractic scope of practice as defined by the French 2011 decree [12, 13].

‘Clarifying explanation’ was defined as text related to a non-MSK condition that revealed whether a website claimed to offer treatment for non-MSK conditions, as opposed to describing non-MSK symptoms as secondary to MSK disorders or vice versa. For example, it was considered to be adequate to claim that patients can have sleeping disorders due to some MSK pain during the night and to claim that MSK back pain can induce dysfunctional breathing or abdominal pain. However, it was not considered to be adequate if there was no clarifying explanation or if the treatment of non-MSK disorders was justified with the *subluxation theory* (i.e., organ dysfunctions could be caused by nerves being compromised in the spinal canals; freeing these nerves—with spinal adjustments [manipulations]—would prevent/treat organs dysfunctions).

After the two training sessions, the data collection group (CC, GG, MP, SP, PF) decided to add two new non-MSK conditions to the data collection tool: ‘Sleep problems among adults’ and ‘Skin disease (other than herpes zoster)’.

### Statistical analysis

The equation used for sample size calculations estimates the finite population corrected sample size for proportions. With a population of 1286 chiropractors, a confidence level of 95% and a margin of error of 5% a sample of 296 (23%) chiropractic websites would reflect the target population.

In order to evaluate the representativeness of the final sample (i.e., included chiropractors), the characteristics (sex, affiliation to French Chiropractic Association, country of education and years since graduation) of the selected but not included chiropractors was compared with the final sample of included chiropractors (i.e., a chiropractor with a website to be analysed) using *t*-test or a non-parametric equivalent for continuous variables and chi-squared test for categorical variables.

The results were presented as descriptive statistics and frequency tables. Pearson’s chi-squared tests were used to evaluate differences in the clinics’ characteristics and the frequency of reporting treatment of non-MSK conditions. In case of significant differences, pairwise (post-hoc) comparisons were performed using chi-square tests for proportions or Fisher’s exact test to identify the specific groups that differed. The statistical significance level was set at 5%. Data management and analysis including sample size calculation were performed using STATA version 16.0 (StataCorp LLC, TX77845, USA).

## Results

### Selection of included chiropractors

**Fig. 1:**
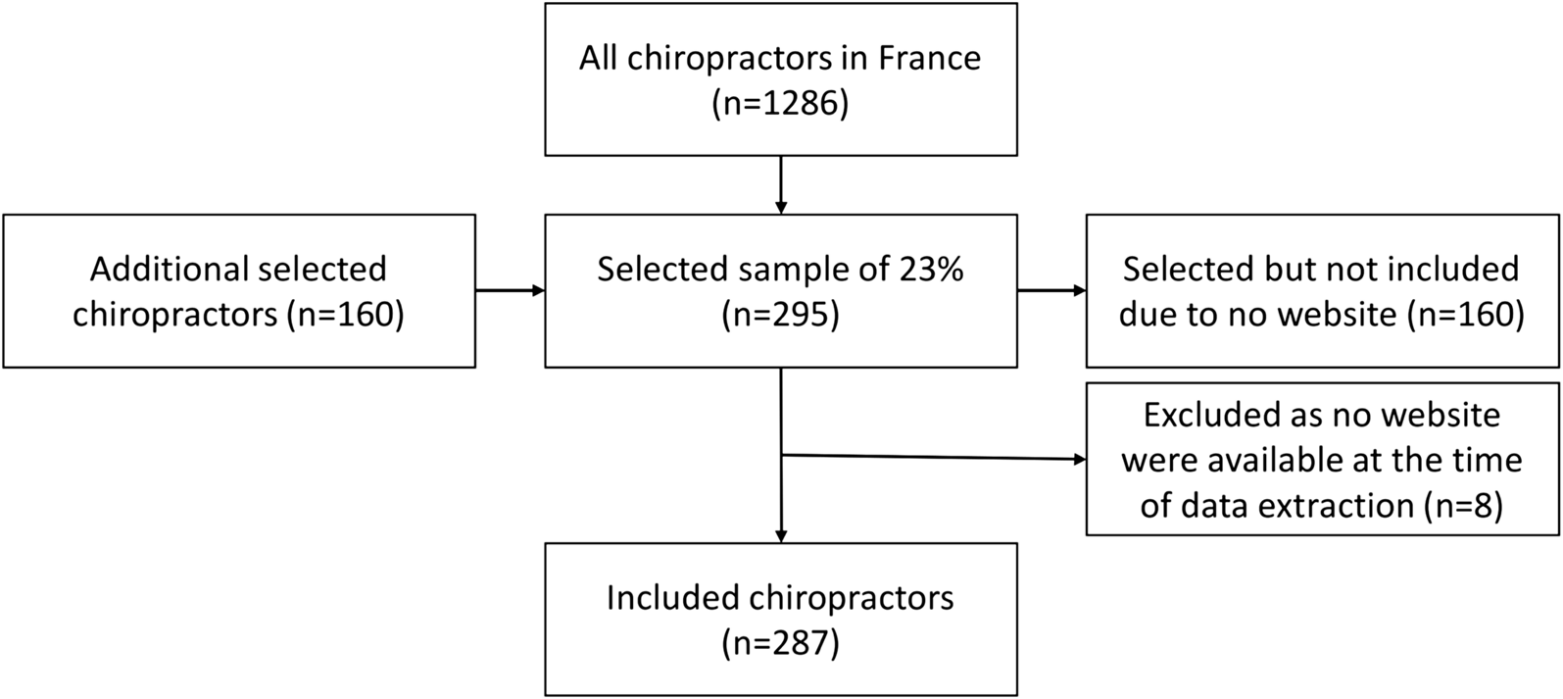
Flowchart describing the selection process of included chiropractors

A total of 1286 chiropractors were registered in France at the time of data collection. After the first random selection, 295 (23%) chiropractors were selected. As many of the selected chiropractors did not have a website, several random selection rounds were required to reach 295 chiropractors with a website. The number of random selection rounds varied between two (region 9) and 18 (region 12). A flowchart (Fig. 1) illustrates the selection process of included chiropractors. A detailed figure describing the selection rounds per region is available in Additional file 2. At the time of data extraction, another eight websites turned out to be unavailable. This was because the website was shut down between the time of selection of websites and the time of data extraction. Consequently, the final number of included websites was 287. Descriptive information and a comparison between included and ‘selected but not included’ chiropractors are presented in Table 1. The ‘included’ group contained more chiropractors educated in France compared to the ‘selected but not included’ group, where chiropractors were more often educated in the USA. Also, a larger proportion of the included chiropractors were members of the AFC compared to the ‘selected but not included’ chiropractors. No other differences were found.

**Table 1:** Characteristics of included and the selected but not included chiropractors

	Included	Selected but not included	Total	P-value	Missing/N (%)
n (%)	287 (63.1)	168 (36.9)	455 (100)		0/455 (0.00)
Region, n (%)					
(1) Auvergne Rhône Alpes	38 (13.2)	13 (7.7)	51 (11.2)		
(2) Haut de France	15 (5.2)	6 (3.6)	21 (4.6)		
(3) Provence Alpes Côtes d’Azur	25 (8.7)	16 (9.5)	41 (9.0)		
(4) Grand Est	18 (6.3)	13 (7.7)	31 (6.8)		
(5) Occitanie	33 (11.5)	20 (11.9)	53 (11.6)		
(6) Normandie	13 (4.5)	8 (4.8)	21 (4.6)		
(7) Nouvelle Aquitaine	35 (12.2)	23 (13.7)	58 (12.7)		
(8) Centre Val de Loire	7 (2.4)	7 (4.2)	14 (3.1)		
(9) Corse	1 (0.3)	1 (0.6)	2 (0.4)		
(10) Bourgogne Franche-Comté	9 (3.1)	1 (0.6)	10 (2.2)		
(11) Pays de la Loire	10 (3.5)	6 (3.6)	16 (3.5)		
(12) Bretagne	12 (4.2)	9 (5.4)	21 (4.6)		
(13) Ile de France	71 (24.7)	45 (26.8)	116 (25.5)	0.71	0/455 (0.00)
Sex, n (%)					
Male	133 (46.3)	76 (45.2)	209 (45.9)		
Female	154 (53.7)	92 (54.8)	246 (54.1)	0.82	0/455 (0.00)
Years in practice, median (IQR)	7.0 (4.8; 15.0)	8.0 (3.0; 23.0)	8.0 (4.0; 17.0)	0.83	34/455 (7.47)
Country of education, n (%)					
France	259 (90.2)	123 (80.4)	382 (86.8)		
USA	25 (8.7)	25 (16.3)	50 (11.4)		
UK	3 (1.0)	5 (3.3)	8 (1.8)	0.01	15/455 (3.30)
Affiliation to the French Chiropractic Association (AFC), n (%)					
No	102 (35.7)	93 (55.4)	195 (43.0)		
Yes	184 (64.3)	75 (44.6)	259 (57.0)	0.00	1/455 (0.22)

Descriptive information about the eight included but unavailable websites are shown in Table 2.

**Table 2:** Descriptive information about eight chiropractors with an unavailable website to evaluate

	Region	Sex	Affiliation to French Chiropractic Association	Country of education	Years since graduation
Chiropractor 1	4	Female	No	France	11
Chiropractor 2	5	Male	No	USA	21
Chiropractor 3	5	Female	No	France	–
Chiropractor 4	12	Male	Yes	France	19
Chiropractor 5	12	Male	No	USA	37
Chiropractor 6	13	Male	No	France	1
Chiropractor 7	13	Female	No	France	5
Chiropractor 8	13	Female	Yes	France	1

Disagreement within pairs of data collectors was resolved through discussion and a second opinion about a website was never required.

No added variables emerged during the data collection process and the predefined list of 44 potential diagnoses and conditions was used for the assessment of the websites.

### Patient groups

The special interest groups most often mentioned on the websites were ‘children’ (n = 251, 87%), ‘pregnant women’ (n = 245, 85%), ‘athletes’ (n = 243, 85%), ‘seniors’ (n = 241, 84%), and ‘infants’ (n = 238, 83%). A total of 215 websites (75%) mentioned all of these five groups. ‘Musicians’ (n = 13, 5%) and ‘disabled people’ (n = 7, 2%) were only rarely mentioned. Finally, ‘animals’ were mentioned on 4% of the websites (n = 12).

### Diagnoses and symptoms of non-musculoskeletal conditions

Of the 44 different diagnoses or symptoms of non-MSK origin on the predefined list, 42 were identified on the websites. One or more of these 42 diagnoses or symptoms were present on 231 (80.5%) of the 287 websites. Of these, 5 (2.2%) websites provided an adequate clarifying explanation for all their non-MSK claims. A total of 226 (78.7%) websites mentioned one or more non-MSK conditions or diagnoses without a clarifying explanation. The non-MSK conditions most often mentioned on the websites were ‘Sleep problems (among adults)’ (n = 153, 68%), ‘Constipation/digestive problems’ (n = 149, 66%), ‘Insomnia/unease/discontent (in children)’ (n = 133, 59%), and ‘Menstrual cramps/pains’ (n = 97, 43%). Detailed information is provided in Table 3.

**Table 3:** Non-musculoskeletal diagnosis and symptoms reported on 287 French websites

Symptoms/diagnosis	Number of websites reporting non-MSK in total (n = 231)	Number of websites reporting non-MSK without an adequate explanation (n = 226)
Sleep problems (among adults)	153	149
Constipation/digestive problems	149	133
Insomnia/unease/discontent (in children)	133	133
Menstrual cramps/pains	97	94
Immune system	79	53
Chronic fatigue syndrome (CFS)	70	70
Concentration/attention problems (in children)	67	67
Otitis media/ear infection (in children)	64	64
Respiratory problems (other than asthma)	62	46
Tinnitus	56	54
Skin disease (other than herpes zoster)	54	54
Hyperactivity/restlessness (in children)	45	45
Problems with suckling/breast-feeding (in children)	40	40
Hormonal imbalance	40	29
Abdominal pain	38	38
Internal organs	38	36
Asthma	37	37
Allergy	28	28
High blood pressure	28	28
Learning problem (in children)	28	28
Incontinence/bed-wetting (in children)	27	27
Sinusitis	25	25
Infection	23	23
Common cold	22	22
Language, reading or writing difficulties	21	21
Changes in mood (in children)	18	18
Nausea	18	18
Low blood pressure	17	17
Impotence	15	15
Irritable bowel syndrome	14	14
Attention-deficit/hyperreactive disorder (in children)	13	13
Vision impairment/disturbance	13	13
Eye and ear pain	11	11
Trigeminal neuralgia	11	11
Highly sensitive children	7	7
Ménière's disease	4	4
Concussion	3	3
Vestibular neuronitis	3	3
Chronic obstructive pulmonary disease	2	2
Shingles (herpes zoster)	2	2
Swelling/bleeding/wounds	2	2
Tumour	1	1
Complex regional pain syndrome 1	0	0
Osteoporosis	0	0

### Difference between chiropractors

No difference was observed in geography (Region), country of educational background, or years since graduation between those chiropractors who advertised treatment for non-MSK disorders and those who did not. However, a larger proportion of chiropractors who were members of the French Chiropractic Association were advertising treatment for non-MSK disorders (84%) compared to those who were not members (69%) (*p* = 0.002).

### Advertising for special interest groups

A larger proportion of websites advertising treatment for non-MSK disorders was found among chiropractors claiming a special clinical focus on infants or children (84%) compared to websites that did not focus on infants or children (40%) (*p* < 0.001). The same pattern was observed for those claiming a special clinical focus on seniors (85%) compared to those not advertising for seniors (48%) (*p* < 0.001), for those claiming a special clinical focus on pregnant women (85%) compared to those who did not (47%) (*p* < 0.001) and for those claiming a special clinical focus on athletes (82%) compared to those who did not (59%) (*p* = 0.001).

## Discussion

### Summary of the results

Of the 287 randomly selected websites, one or more non-MSK claims were identified on almost 8 out of 10 websites. The non-MSK conditions most often mentioned on the websites were sleep problems (among children and adults), constipation/digestive problems and menstrual cramps/pains. A larger proportion of chiropractors who were members of the French Chiropractic Association (AFC) were advertising treatment for non-MSK disorders compared to those who were not members. A larger proportion of websites advertising treatment for non-MSK disorders was found among chiropractors claiming a special clinical focus on infants/children, seniors, pregnant women or athletes compared to websites that did not focus on these special interest groups.

### Comparison with previous studies

A study by Ernst and Gibley [22] investigated the frequency of claims to treat some non-MSK conditions, including asthma and ear infection/otitis media/earache, by chiropractors in the English-speaking world. The study found that among 200 chiropractic websites (convenience sample), 52% reported treating asthma compared to 13% (37/287) in our study and 55% reported treating ear infection/otitis media/earache compared to 22% for ‘otitis media/ear infection in children’ and 4% for ‘eye and ear pain’ in our study. Another study by Murdoch et al. [18] investigated claims on diagnosis, treatment and/or efficacy for allergy and asthma by some Canadian complementary and alternative medicine practitioners (including chiropractors). The study found that among 100 chiropractic websites, 33% claimed diagnosis, treatment and/or efficacy for allergy/sensitivity and 38% for asthma. By comparison, ‘allergy and asthma’ was mentioned by 10% (28/287) and 13%, respectively, in the present study. Compared to the two studies mentioned above, French chiropractors seem to be less likely to mention diagnoses like asthma and allergy. However, 78.7% still mentioned one or more non-MSK conditions but these claims were more often related to symptoms rather than specific diagnoses. Sleep problems, constipation/digestive problems and menstrual cramps/pains were the non-MSK conditions most often mentioned in the present study, and they were all related to unspecific symptoms rather than specific diagnoses.

The method used in the present study is comparable to a recent cross-sectional study by Jensen et al. [16] investigating website claims by Danish chiropractors. In the present study, a larger proportion of chiropractors (78.7%) mentioned diagnoses or symptoms of non-MSK origin without an acceptable clarifying explanation compared to the Danish study (25.9%). Six non-MSK conditions were reported by 20% or more of all chiropractic clinics in both studies: Abdominal pain; Concentration/attention problems (in children); Constipation/digestive problems; Hyperactivity/restlessness (in children); Insomnia/unease/discontent (in children); Otitis media/ear infection (in children).

Except for otitis media, all these conditions are related to symptoms or syndromes rather than specific diagnoses. Also, most of them are relevant for children. It is important to specify that these results only provide a general view on what is advertised on chiropractic websites. The results do not identify what chiropractors treat in their practice. We also do not know what the chiropractors specifically meant by what they wrote or what the patients understand from reading these claims.

The data collection group identified that a standardized website, which included several of the non-MSK claims listed on the collection tool, was used by a large proportion of the included chiropractors. This website corresponds to an old version of the AFC website created by a private company. The company and AFC engaged in co-operation to offer preferential fees and assistance for chiropractors who wanted a professional website. The assistance included the use of pre-written texts. It is possible that this could help explain why a larger proportion of chiropractors who were a member of the French Chiropractic Association were advertising treatment for non-MSK disorders. If this is the case, it would illustrate the impact that a professional association can have on its members.

### Methodological considerations

Websites were selected from all regions, ensuring that the findings include the ‘underrepresented’ smaller regions (e.g., regions 6, 8, 9 and 10). Several random selections were made to ensure that the included chiropractors were randomly chosen. This ensured that the final sample included in this study was representative of all chiropractors with a website. With a CI of 95% and a margin of error of 5%, we estimated that 296 websites would reflect the target population of 1286. However, only 287 websites were included which corresponds to a CI of 95% and a margin of error of 5.1% which we consider acceptable.

The creation of the French data collection tool was supervised by a senior researcher who was bilingual in Danish and French and who has lived and worked in both counties. This reduced the risk of linguistic or cultural errors when adapting the tool to a French context. One member of the data collection team reviewed all of the websites to ensure the best possible homogeneity between the evaluation teams. However, although an agreement was reached without involving a third person, it is not possible to avoid subjectivity when assessing if the clarifying explanation was adequate or not if non-MSK disorders were mentioned.

### Perspectives

This study examined website claims by extracting and interpreting information provided on the websites. However, it is not clear what the chiropractor intended by providing the information. It is possible that the wording is inaccurate, the focus on non-MSK conditions was unintentional or the website was neglected. To better understand the intentions of the website claims it is necessary to conduct qualitative studies or explorative surveys. Also, some websites leave an impression of pseudo-scientific tendencies, for example (i) absence of non-indication to chiropractic treatment; (ii) testimonials as proof of efficacy; (iii) extraordinary claims; or (iv) argumentation based on historical chiropractic theories. These types of claims should be considered for inclusion in future studies in this area of research.

To our knowledge, no similar study has been conducted among other health care providers who work with manual therapies (e.g., physiotherapists/*kinésithérapeutes,* osteopaths) in France. It is possible that well-regulated health care providers (e.g., physiotherapists/*kinésithérapeutes*) would be less inclined to write unsupported claims on their websites, compared with health care providers who are less regulated (e.g., chiropractors, osteopaths). It would therefore be relevant to investigate the communication of these professionals and to compare the results with the respective level of recognition of these professionals in the healthcare system.

In chiropractic, non-MSK conditions are not a predominant reason for patients seeking care [11], and treatment of non-MSK conditions is not in accordance with the latest scientific consensus as per a recent systematic review [7] which did not find evidence of an effect of SMT on the management of several non-MSK disorders (e.g., childhood asthma, hypertension, primary dysmenorrhea). Mentioning non-MSK conditions on professional chiropractic websites can mislead patients toward non-useful treatments or even delay relevant medical care. It can also be prejudicial for the reputation of the chiropractic profession as a whole and thereby unintentionally delay or obstruct integration into the national health care systems. On a small scale, professional chiropractic bodies could provide clear guidelines on advertisement for chiropractors and encourage critical review of existing websites. However, to truly change the chiropractic scope of practice towards an evidence-based paradigm, it would require a much bigger effort involving education systems, national health care systems, professional bodies, and experts in the field of manual therapies as well as implementation and behaviour change research.

## Conclusions

In a random sample of 287 chiropractic websites in France, 78.7% mentioned one or more non-MSK diagnoses or symptoms. It is unclear if this reflects unclear communication or if treatments for non-MSK conditions are provided by the chiropractors. Either way, it could be misleading to patients and is not in agreement with the French legislation.

## Supplementary Information

**Additional file 1**. English version of the data collection tool

**Additional file 2**. Flowchart describing, for each random selection (Rand.), the number of selected and included chiropractors (included/selected)

## Data Availability

The data used for the current study are available from the corresponding author on reasonable request.

## Abbreviations

AFC: French Chiropractic Association (*Association Française de Chiropraxie*)
MSK: Musculoskeletal
SMT: Spinal Manipulative Therapy
UK: United Kingdom
USA: United States of America

## References

[1] Sackett DL, Rosenberg WM, Muir Gray AJ, Brian H, Aynes R, Richardson WS (1996). Evidence based medicine: what it is and what it isn’t. BMJ.

[2] Kjaer P, Kongsted A, Hartvigsen J, Isenberg-Jørgensen A, Schiøttz-Christensen B, Søborg B (2017). National clinical guidelines for non-surgical treatment of patients with recent onset neck pain or cervical radiculopathy. Eur Spine J.

[3] Coulter ID, Crawford C, Hurwitz EL, Vernon H, Khorsan R, Suttorp Booth M (2018). Manipulation and mobilization for treating chronic low back pain: a systematic review and meta-analysis. Spine J.

[4] Stochkendahl MJ, Kjaer P, Hartvigsen J, Kongsted A, Aaboe J, Andersen M (2018). National clinical guidelines for non-surgical treatment of patients with recent onset low back pain or lumbar radiculopathy. Eur Spine J.

[5] Foster NE, Anema JR, Cherkin D, Chou R, Cohen SP, Gross DP (2018). Prevention and treatment of low back pain: evidence, challenges, and promising directions. Lancet.

[6] Coulter ID (2019). Manipulation and mobilization for treating chronic nonspecific neck pain: a systematic review and meta-analysis for an appropriateness panel. Pain Phys.

[7] Côté P, Hartvigsen J, Axén I, Leboeuf-Yde C, Corso M, Shearer H (2021). The global summit on the efficacy and effectiveness of spinal manipulative therapy for the prevention and treatment of non-musculoskeletal disorders: a systematic review of the literature. Chiropr Man Ther.

[8] Bronfort G, Haas M, Evans R, Leininger B, Triano J (2010). Effectiveness of manual therapies: the UK evidence report. Chiropr Osteopat.

[9] Clar C, Tsertsvadze A, Court R, Hundt GL, Clarke A, Sutcliffe P (2014). Clinical effectiveness of manual therapy for the management of musculoskeletal and non-musculoskeletal conditions: systematic review and update of UK evidence report. Chiropr Man Ther.

[10] Goncalves G, Le Scanff C, Leboeuf-Yde C (2018). Effect of chiropractic treatment on primary or early secondary prevention: a systematic review with a pedagogic approach. Chiropr Man Ther.

[11] Beliveau PJH, Wong JJ, Sutton DA, Simon NB, Bussières AE, Mior SA (2017). The chiropractic profession: a scoping review of utilization rates, reasons for seeking care, patient profiles, and care provided. Chiropr Man Ther.

[12] Légifrance. Loi n° 2002–303 du 4 mars 2002 relative aux droits des malades et à la qualité du système de santé (1). https://www.legifrance.gouv.fr/loda/article_lc/LEGIARTI000023857681/2011-10-06/. Accessed 7 Feb 2021.

[13] Légifrance. Décret n° 2011–32 du 7 janvier 2011 relatif aux actes et aux conditions d’exercice de la chiropraxie. https://www.legifrance.gouv.fr/affichTexte.do?cidTexte=JORFTEXT000023387301. Accessed 9 Apr 2020.

[14] Légifrance. Arrêté du 13 février 2018 relatif à la formation en chiropraxie. http://solidarites-sante.gouv.fr/fichiers/bo/2018/18-02/ste_20180002_0000_0099.pdf. Accessed 9 Apr 2020.

[15] Légifrance. Décret no 2020–1662 du 22 décembre 2020 portant modification du code de déontologie des médecins et relatif à leur communication professionnelle. https://www.legifrance.gouv.fr/jorf/id/JORFTEXT000042731060. Accessed 7 Feb 2021.

[16] Jensen RK, Agersted MEI, Nielsen HA, O’Neill S (2020). A cross-sectional study of website claims related to diagnoses and treatment of non-musculoskeletal conditions. Chiropr Man Ther.

[17] Shelley J, Clark M, Caulfield T (2015). The face of chiropractic: evidence-based?: original article. Focus Altern Complement Ther.

[18] Murdoch B, Carr S, Caulfield T (2016). Selling falsehoods? A cross-sectional study of Canadian naturopathy, homeopathy, chiropractic and acupuncture clinic website claims relating to allergy and asthma. BMJ Open.

[19] Hanna M, Honeychurch M (2016). Chronic misleading online advertising by chiropractors. NZ Med J.

[20] Havemann DD. South African chiropractic website claims related to diagnosis and treatment of non-musculoskeletal conditions: a cross-sectional study. 137.

[21] Random Generator Number. https://www.random.org.

[22] Ernst E, Gilbey A (2010). Chiropractic claims in the English-speaking world. NZ Med J.

