# Supplementary material for: Chiropractic website claims related to non-musculoskeletal conditions: a cross-sectional study
Source: Chiropr Man Therap. 2021 Sep 22;29:39. doi: 10.1186/s12998-021-00397-y (PMC8456627; doi:10.1186/s12998-021-00397-y)
Supplement: Supplementary file 1 — Additional file 1. English version of the data collection tool [file 12998_2021_397_MOESM1_ESM.docx]

**Additional file 1. English version of the data collection tool**

| Data collection tool (adapted from the Danish version) |  |  |
| --- | --- | --- |
| ID number |  |  |
| Affiliation do AFC (0=no, 1=yes) |  |  |
| Region (1=North, 2=Central, 3= South etc…) |  |  |
| Interest or specialist focus |  |  |
| Advertisement for treating infants (0=no, 1=yes) |  |  |
| Advertisement for treating children (0=no, 1=yes) |  |  |
| Advertisement for treating seniors (0=no, 1=yes) |  |  |
| Advertisement for treating pregnant women (0=no, 1=yes) |  |  |
| Advertisement for treating athletes (0=no, 1=yes) |  |  |
| Advertisement for treating disabled people (0=no, 1=yes) |  |  |
| Diagnosis and symptoms | Present on website  (0=no, 1=yes) | If present, MSK based explanation  (0=no, 1=yes) |
| Abdominal pain |  |  |
| Allergy |  |  |
| Asthma |  |  |
| Attention-deficit/hyperreactive disorder (in children) |  |  |
| Changes in mood (in children) |  |  |
| Chronic fatigue syndrome (CFS) |  |  |
| Chronic Obstructive Pulmonary Disease |  |  |
| Common cold |  |  |
| Complex regional pain syndrome (Type 1) |  |  |
| Concentration problems (in children) |  |  |
| Concussion |  |  |
| Constipation/digestive problems |  |  |
| Eye and ear pain |  |  |
| High blood pressure |  |  |
| Highly sensitive children |  |  |
| Hormonal imbalance |  |  |
| Hyperactivity/restlessness (in children) |  |  |
| Immune system |  |  |
| Impotence |  |  |
| Incontinence/bed-wetting (in children) |  |  |
| Infection |  |  |
| Insomnia/unease/discontent (in children) |  |  |
| Internal organs |  |  |
| Irritable bowel syndrome |  |  |
| Language, reading or writing difficulties |  |  |
| Learning difficulties (in children) |  |  |
| Low blood pressure |  |  |
| Ménière's disease |  |  |
| Menstrual cramps/pains |  |  |
| Nausea |  |  |

| Diagnosis and symptoms | Present on website  (0=no, 1=yes) | If present, MSK based explanation  (0=no, 1=yes) |
| --- | --- | --- |
| Osteoporosis |  |  |
| Otitis media/ear infection (in children) |  |  |
| Problems with suckling/breast-feeding (in children) |  |  |
| Respiratory problems (other than asthma) |  |  |
| Shingles (herpes zoster) |  |  |
| Sinusitis |  |  |
| Swelling/ bleeding/wound |  |  |
| Tinnitus |  |  |
| Trigeminal neuralgia |  |  |
| Tumour |  |  |
| Vestibular neuronitis |  |  |
| Vision impairment/disturbance |  |  |
