# Supplementary material for: Chiropractic website claims related to non-musculoskeletal conditions: a cross-sectional study
Source: Chiropr Man Therap. 2021 Sep 22;29:39. doi: 10.1186/s12998-021-00397-y (PMC8456627; doi:10.1186/s12998-021-00397-y)
Supplement: Supplementary file 2 — Additional file 2. Flowchart describing, for each random selection (Rand.), the number of selected and included chiropractors (included/selected) [file 12998_2021_397_MOESM2_ESM.docx]

**Additional file 2. Flow-chart describing, for each random selection (Rand.), the number of selected and included chiropractors (included/selected)**

…

Rand.

18

Rand.

1

Rand.

2

Rand.

3

Rand.

4

Rand.

5

Rand.

6

Rand.

7

Rand.

8

Rand.

9

Rand.

10

…

32/74

27/42

7/15

6/8

0/2

2/2

-

-

-

-

-

1/1

0/1

0/1

1/2

0/2

1/3

0/3

1/4

1/5

2/7

7/14

4/10

3/6

0/3

2/3

1/1

-

-

-

-

-

-

8/9

0/1

1/1

-

-

-

-

-

-

-

-

0/1

1/1

-

-

-

-

-

-

-

-

-

-

-

-

-

-

1/1

0/1

0/1

2/3

4/7

0/7

12/35

9/23

4/14

6/10

3/4

0/1

1/1

-

-

-

-

5/13

2/8

2/6

2/4

1/2

0/1

0/1

0/1

1/1

-

-

17/35

6/18

6/12

2/6

1/4

0/3

1/3

0/2

2/2

-

-

8/19

5/11

5/6

1/1

-

-

-

-

-

-

-

-

-

-

1/1

0/1

0/1

3/4

3/7

5/12

4/16

9/25

-

-

2/2

0/2

0/2

0/2

0/2

1/3

2/5

1/6

9/15

27/38

5/11

3/6

1/3

0/2

2/2

-

-

-

-

-

Region 1

Region 2

Region 3

Region 6

Region 7

Region 10

Region 11

Region 12

Region 9

Region 5

Region 4

Region 8

Region 13
